# Identification of PDL1-Related Biomarkers to Select Lung Adenocarcinoma Patients for PD1/PDL1 Inhibitors

**DOI:** 10.1155/2020/7291586

**Published:** 2020-06-09

**Authors:** Yanping Wu, Lianjun Lin, Xinmin Liu

**Affiliations:** Department of Geriatrics, Peking University First Hospital, Beijing 100034, China

## Abstract

PD1/PDL1 inhibitors have been adopted for the treatment of advanced non-small-cell lung cancer, and PDL1 expression has been investigated as a predictive biomarker for PD1/PDL1 inhibitor therapy. However, PDL1 lacks diagnostic accuracy in differentiating patients who are likely or unlikely to benefit. So, it is urgent and clinically significant to identify other associated predictive biomarkers for PD1/PDL1 inhibitor therapy. Our work was to identify PDL1-related biomarkers that could improve the patient selection for PD1/PDL1 inhibitor treatment. We obtained 500 genes coexpressed with PDL1 in lung adenocarcinoma from the TCGA database. Then, we identified 125 out of 500 genes differentially expressed in lung adenocarcinoma. A total of 39 genes were distinguished with prognostic value and associated with overall survival. Median survival time analysis based on gene expression level, protein-protein interaction analysis, GO and KEGG enrichment analyses, and significant GO and KEGG function consistency analyses were conducted to screen candidate biomarkers. Three candidate genes, BRCA1, BRIP1, and EREG, were identified to be functionally significantly coexpressed with PDL1. Functional enrichment analysis and protein-protein interaction networks further showed that these genes mainly participated in immune response and cell activation. Additionally, to find potential adjuvant therapeutic targets in PD1/PDL1 inhibitor treatment, we performed transcription factor prediction analysis. A group of negative differential expression but PDL1-related biomarkers has been identified, which might help to assess the clinical management of lung cancer patients. A combination of potential biomarkers and adjuvant therapeutic targets with PDL1 will predict the response to PD1/PDL1 inhibitors more accurately and help with the patient selection for more personalized immune checkpoint inhibitor treatment.

## 1. Introduction

Lung cancer is a leading cause of cancer death worldwide due to its low survival rate [1, 2]. Non-small-cell lung cancer (NSCLC) accounts for 85% of lung cancer, and lung adenocarcinoma is the most common histological type of NSCLC [3]. Traditional therapeutic options remain limited for patients, and recently, immunotherapy emerges and becomes popular because of its outstanding efficacy [4]. PD1/PDL1 immune checkpoint inhibitors have been developed and adopted for the treatment of NSCLC. PD1 is expressed by activated T cells, B lymphocytes, and natural killer cells. PDL1 was identified as PD1 ligand. PDL1 is expressed by T lymphocytes, epithelial cells, endothelial cells, tumor cells, and other cells in the local tumor environment. The interaction between PD1 and PDL1 suppresses T cell activation and helps tumor cells to escape immune surveillance.

Nowadays PD1/PDL1 immune checkpoint inhibitors, including nivolumab, pembrolizumab, atezolizumab, and durvalumab, have been adopted to treat NSCLC [5, 6]. A study revealed that pembrolizumab monotherapy has shown significant improvements in overall survival as a first-line treatment compared with conventional chemotherapy for locally advanced or metastatic non-small-cell lung cancer without sensitising EGFR or ALK alterations when PDL1 TPS ≥ 1% [7]. Pembrolizumab monotherapy now is recommended as a first-line therapy for patients with NSCLC without driver gene mutations and with a high PDL1 expression (tumor proportion score (TPS) ≥ 50%). Nivolumab or atezolizumab is recommended in a second-line setting for NSCLC regardless of PDL1 expression. A multicentre, randomised, open-label, phase 3 trial revealed that the treatment benefit was observed in terms of overall survival and progression-free survival in the subgroup populations when atezolizumab in combination with carboplatin plus nab-paclitaxel chemotherapy as the first-line treatment for metastatic nonsquamous non-small-cell lung cancer, regardless of PDL1 expression [8]. Atezolizumab is now FDA approved in the first-line setting in combination with carboplatin, paclitaxel, and bevacizumab for patients with metastatic nonsquamous NSCLC with no EGFR or ALK genomic tumor mutations. Regarding locally advanced NSCLC, durvalumab currently represents the only FDA-approved and recommended immune checkpoint inhibitor for the treatment of unresectable stage III NSCLC patients, irrespective of histological type and PDL1 expression, whose disease has not progressed after a previous chemoradiotherapy treatment [9]. However, according to a recent meta-analysis, among patients with PDL1 expression < 1%, docetaxel monotherapy second-line treatment was not superior to immune checkpoint inhibitors [10].

At present, PDL1 is the only predictive biomarker validated for the selection of patients who could benefit from pembrolizumab, and PDL1 expression in tumor cells is considered to be prognostic in NSCLC. However, so far, there is no consensus in defining the PDL1 expression level as positive or negative (ranging from 1% to 50% expression) [11]. And there is significant intratumor heterogeneity for the PDL1 expression, and a biopsy may not be representative of the entire tumor mass [12]. Another debate focus is whether immunotherapy is active in patients with NSCLC who have an activating genetic abnormality such as EGFR mutation or ALK translocation. Then, the inevitable drug resistance issue and the mechanisms of resistance are currently poorly understood. Given the controversial results and important drawbacks, it is important to find other potential, reliable biomarkers in combination with PDL1 to improve the selection of patients for PD1/PDL1 inhibitor treatment.

## 2. Materials and Methods

### 2.1. Patients and Samples

The mRNA sequencing data and related clinical information of 533 lung adenocarcinoma tissue samples and 59 adjacent nontumor tissue samples were obtained from The Cancer Genome Atlas (TCGA) database. Patients who had other malignancies than lung adenocarcinoma were excluded. As the data were retrieved from TCGA database, data processing procedures in this study met the guideline of the TCGA human subject protection and data access policies.

### 2.2. Differential Expression Analysis

We performed differential expression analysis by comparing mRNA expression in human lung adenocarcinoma tissues and adjacent normal tissues. The differential expression of mRNAs between human lung adenocarcinoma tissues and adjacent nontumor tissues were compared using the DESeq2 R package with Wald significance tests. The DESeq2 model and all the steps taken in the software were described in a previous publication [13], and we include the formula and descriptions in this section as well. The differential expression analysis in DESeq2 uses a generalized linear model of the form:

(1) $$\begin{array}{c} K_{ij}\text{\textasciitilde{}NB}\left(\mu_{ij},\alpha_{i}\right),\\ \mu_{ij}=s_{j}q_{ij},\\ \log_{2}\left(q_{ij}\right)=x_{j.}\beta_{i}, \end{array}$$

where counts *K*_*ij*_ for gene *i*, sample *j* are modeled using a negative binomial distribution with fitted mean *μ*_*ij*_ and a gene-specific dispersion parameter *α*_*i*_. The fitted mean is composed of a sample-specific size factor *s*_*j*_ and a parameter *q*_*ij*_ proportional to the expected true concentration of fragments for sample *j*. The coefficients *β*_*i*_ give the log_2_ fold changes for gene *i* for each column of the model matrix *x*. Note that the model can be generalized to use sample- and gene-dependent normalization factors *s*_*ij*_. *P* values less than 0.05 were considered statistically significant.

### 2.3. Coexpression Analysis

We generated mRNAs-PDL1 coexpression network based on the human lung adenocarcinoma-associated genes in TCGA by the cor function of R package. Significant correlation pairs were used to construct the network based on the Pearson correlation coefficients and the *P* values. The annotation of protein cellular localization and biological function was performed by using the protein-protein interaction (PPI) network. The PPI network was retrieved from the STRING database and reconstructed via Cytoscape software. To assess the main function of the PDL1-associated genes, Gene Ontology (GO) and Kyoto Encyclopedia of Genes and Genomes (KEGG) analyses were performed using the ClusterProfile of R package with the hypergeometric distribution test.

(2) $$\begin{array}{c} p=1-\overset{k-1}{\underset{i=0}{\sum }}\frac{\left(\begin{array}{c} M\\ i \end{array}\right)\left(\begin{array}{c} N-M\\ n-i \end{array}\right)}{\left(\begin{array}{c} N\\ n \end{array}\right)}. \end{array}$$

In this equation, *N* is the total number of genes in background distribution, *M* is the number of genes within that distribution that are annotated (either directly or indirectly) to the gene set of interest, *n* is the size of the list of genes of interest, and *k* is the number of genes within that list which are annotated to the gene set. *P* value < 0.05 was set as the cutoff criterion.

### 2.4. Survival Analysis

We performed survival analysis to further investigate whether the coexpressed genes were associated with the prognosis. Genes were divided into two groups according to the median of gene expression. The gene expression was labeled as high or low using the dichotomy method to determine which gene could potentially be of functional significance in lung adenocarcinoma prognosis. Univariate Cox regression analysis was performed to identify survival-related genes. Kaplan-Meier survival analysis with a log-rank test was performed to compare the differences in overall survival between the high-expression group and low-expression group. The survival curve is calculated by survival R package and plotted by surfminer. The survival probability is calculated by the survival time, survival state, and different grouping conditions, as follows:

(3) $$\begin{array}{c} {\hat{S}}_{KM}\left(t\right)=\underset{s<t}{\prod }\frac{\bar{Y}\left(ts\right)-t\left(s\right)}{\bar{Y}\left(s\right)}. \end{array}$$

Graphically, the Kaplan-Meier survival curve appears as a step function with a drop at each death. Censoring times are often marked on the plot as \+” symbols. KM curves are created with the survfit function. The left-hand side of the formula will be a Surv object and the right-hand side contains one or more categorical variables that will divide the observations into groups.

### 2.5. Transcription Factor Binding Site Prediction

We performed transcription factor binding site (TFBS) prediction analysis of the interest genes. Besides, published data of genes and their transcription factors were collected, we also used http://www.tfbss.org/search/ method to predict the transcription factors of target genes.

## 3. Results

### 3.1. Identification of Differentially Expressed mRNAs in Human Lung Adenocarcinoma

**Figure 1:**
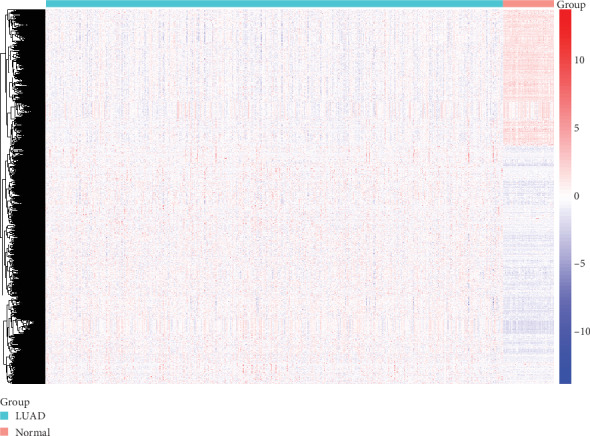
Differentially expressed genes in lung adenocarcinoma. A heat map is showing the differentially expressed mRNAs.

To identify significantly differentially expressed mRNAs, we initially performed mRNA differential expression analysis in lung adenocarcinoma and adjacent normal tissues. A total of 533 lung adenocarcinoma samples and 59 normal samples were obtained from TCGA database. We set fold change > 2 and *P* value < 0.05 as cutoffs to screen significantly differentially expressed mRNAs. Ultimately, a total of 5439 differentially expressed mRNAs were identified, as shown in Figure 1. Among the differentially expressed genes, 3456 genes were upregulated and the rest of the genes were downregulated.

### 3.2. Coexpression of Lung Adenocarcinoma Genes and PDL1

**Figure 2:**
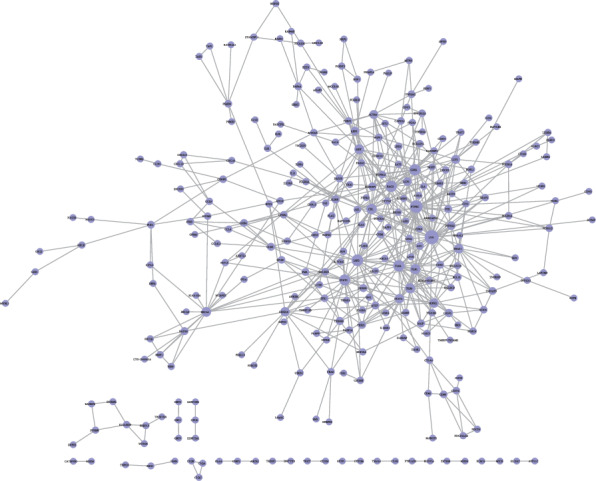
The map represents the protein-protein interaction network of PDL1 coexpressed genes. Nodes represent genes and lines connecting genes represent interactions.

To identify coexpressed genes, we assessed the correlation between PDL1 and lung adenocarcinoma-associated genes in TCGA database. We obtained the top 500 genes when the Pearson correlation coefficient > 0.308571756 and <-0.309493137. Scatter plot showing the coexpression pattern of each lung adenocarcinoma gene and PD-L1 was conducted. Among those genes, when *P* values < 0.05 and fold change > 2 as cutoffs, 125 genes were identified not only coexpressed with PDL1 but also significantly differentially expressed in lung adenocarcinoma. To find out whether those genes identified from TCGA database are also of prognostic significance, we generated the Kaplan-Meier survival curves to explore the potential roles of those genes in overall survival. Among the candidate genes identified in lung adenocarcinoma, a total of 39 genes were validated having a significant effect on the overall survival of patients in the log-rank test (*P* < 0.05, all genes were listed in Table 1, two were approximately equal to 0.05 and were excluded as candidates in the following analysis). To assess the prognostic value of the identified biomarkers (by median), 6 genes were excluded according to the survival time two more weeks less than that of PDL1 as a biomarker. To better understand the interplay among the identified coexpressed genes, we obtained the protein-protein interaction (PPI) network using the STRING tool, as shown in Figure 2. Nine genes that not interacted with others were excluded.

### 3.3. Function Assessment

**Figure 3:**
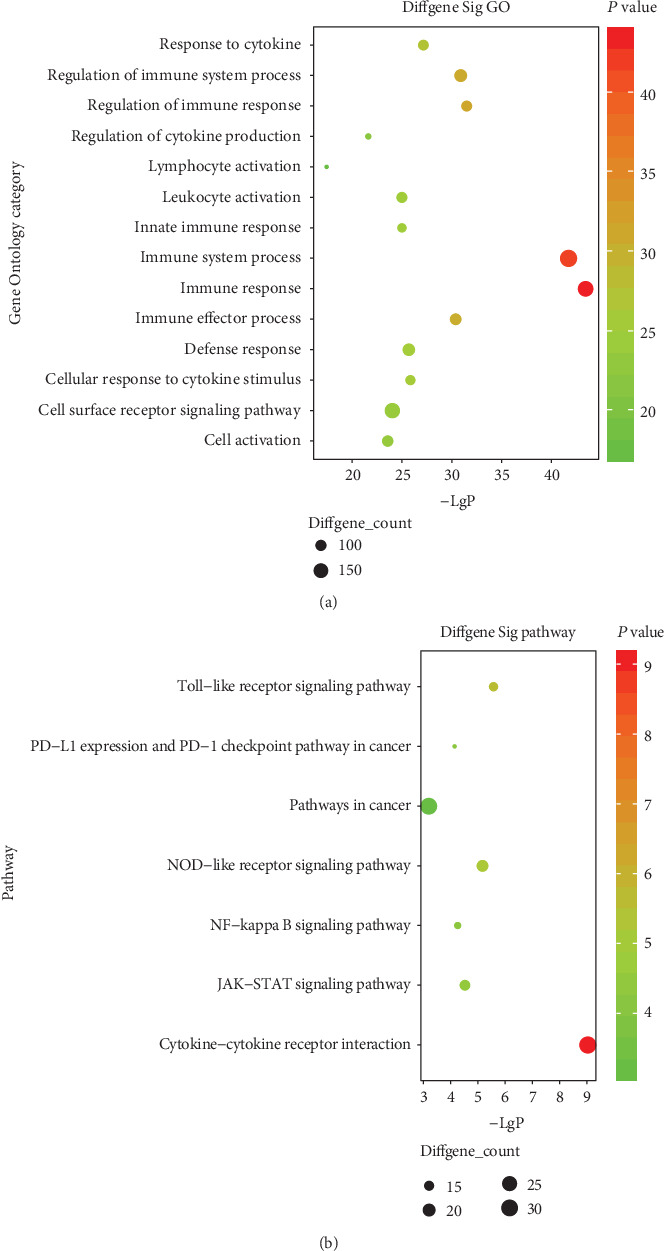
Top 14 enrichment of GO terms and top 7 enrichment of KEGG pathways for 39 candidate genes.

**Figure 4:**
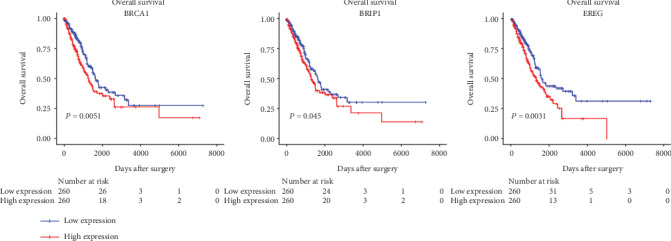
Three candidate genes of significant prognostic value. Kaplan-Meier curves showing the relationship between the three mRNAs and overall survival. The cases were divided into the low- and high-expression groups by the median expression level of genes.

Furthermore, to understand the biological functions and processes these genes were involved in, GO and KEGG enrichment analyses were conducted. Most of the PDL1-associated mRNAs in the coexpression network could be assigned to functional classes related to immune functions and cancer-related pathways. This process revealed enrichment of 45 KEGG pathways (*P* value < 0.05 and enrichment score > 2) and 402 GO terms (*P* value < 0.05 and enrichment score > 10). Five of the top 15 pathways were immune-related pathways including natural killer cell-mediated cytotoxicity, Toll-like receptor signaling pathway, B cell receptor signaling pathway, PDL1 expression and PD1 checkpoint pathway in cancer, and T cell receptor signaling pathway. Top GO terms were Toll-like receptor 2 signaling pathway, positive regulation of gamma-delta T cell activation, cellular response to interferon-beta, regulation of response to tumor cell, and regulation of immune response to tumor cell. Go and KEGG enrichment analyses of 39 candidate genes was shown in Figure 3. Six genes, BRCA1, BRIP1, CSF2RB, CYBB, and EREG, TLR4, were identified according to the consistency of significant GO function and signaling pathway. Eventually, BRCA1, BRIP1, and EREG were chosen as the candidates based on the clinical research progress through searching the PubMed database. Median survival time of patients in the PDL1 high-expression group was 701 days. Median survival time of the identified biomarkers, BRCA1, BRIP1, and EREG, were 690, 701, and 690 days separately. Kaplan-Meier survival curve of three candidate genes was shown in Figure 4.

### 3.4. Possible Adjuvant Therapeutic Targets Based on Transcription Factor Prediction

To identify the possible adjuvant therapeutic targets based on transcription factor prediction, we performed transcription factor prediction analysis. Transcription factor prediction analysis was applied to 152 significantly overlapped genes that were recognized by GO and KEGG enrichment intersection analyses. Statistically significant top 10 predictions of EREG, BRCA1, and BRIP1 were listed, as shown in Table 2.

### 3.5. Possible Adjuvant Biomarkers for Patient Selection Based on Nondifferentially Expressed Genes

**Figure 5:**
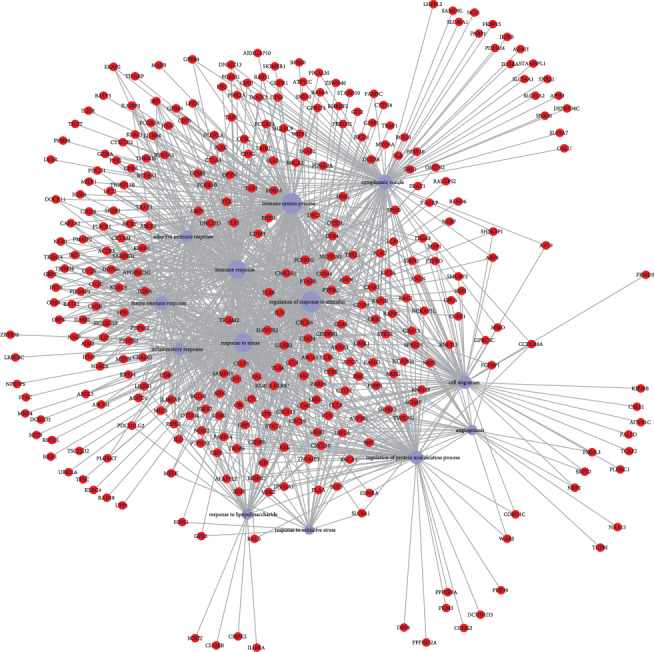
A mRNA-GO network mainly focus on thirteen GO terms. Blue nodes represent GO terms, red nodes represent genes, and lines represent interactions.

To identify other possible adjuvant biomarkers for patient selection, we focus on the 5476 nondifferentially expressed genes. Among the top 500 PDL1-correlated genes, only 114 genes have a negative differential expression in cancer tissues compared with normal tissues. The number of genes that correlated with the overall survival was 21. Eventually, 4 genes, CCR5, FOSL1, NAIP, and NBN, stand out depending to the consistency of significant GO function and signaling pathway and PPI network. Thirteen genes were excluded without the consistency of significant GO function and signaling pathway, four genes were excluded with no interaction in the PPI network. They play a role in immune response, response to stress, and so on. A mRNA-GO network including this group of genes was shown in Figure 5.

## 4. Discussion

Nowadays, the predictive biomarkers for PD1 and PDL1 immune checkpoint blockade therapy are a hot topic. Predictive biomarkers for treatment selection can improve efficiency and reduce costs while protecting patients from hazards of unnecessary treatment [14]. However, there is an unmet need for biomarkers that will identify patients more likely to respond to PD1/PDL1 blockade as well as other immunotherapeutics [15]. The PDL1 expression in combination with other potential biomarkers might get a higher predictive value for response to immune checkpoint inhibitors than the use of an individual biomarker.

In the present study, we sought to identify PDL1-related genes that contribute to the selection of lung adenocarcinoma patients for PD1/PDL1 inhibitor treatment in TCGA database. Treatment details of this set of samples were not available, so the samples diagnosed before April 22, 2014 was screened. Therefore, the patient had a small chance receiving PD1/PDL1 inhibitor therapy, which is a factor we need to consider in identifying candidate biomarkers. First, we identified 3 differential expression genes, EREG, BRCA1, and BRIP1, by comparing median survival time of the identified biomarkers and PDL1, constructing the coexpressed gene PPI network, ensuring consistency between function and signaling pathway, and searching progress in clinical application. Then, to find potential adjuvant therapeutic targets in immune checkpoint treatment, we performed transcription factor prediction analysis and focused on the nondifferentially expressed genes. With further exploration and validation, this observation might contribute to the lung adenocarcinoma patient selection for PD1/PDL1inhibitor therapy.

BRCA1 plays an important role in DNA repair and maintaining genomic stability, and it can also acts as tumor suppressor. BRIP1 is a member of the DEAH helicase family and it binds directly to the BRCT repeats of BRCA1. BRIP1 is a physiological partner of BRCA1, and BRIP1/BRCA1 complex formation contributed to the key activity of BRCA1 [16]. BRCA1 is wildly studied in NSCLC because of its role in chemotherapy response. Taron et al. revealed that BRCA1 could be a predictor for differential chemosensitivity and personal chemotherapy in lung cancer [17, 18]. It was pointed out that no statistically significant correlation was found in BRCA1 expression in NSCLC regarding gender, age, histological type, or smoking status [19–21]. A study by Joerger et al. found that BRCA1 expression differs regarding gender in advanced NSCLC, but not age, histological type, pathological stage, or smoking status [22]. Results also showed that BRCA1 correlated with survival due to its role in chemotherapy response. Recently, there are a variety of clinical trials focusing on BRCA1 as a biomarker to provide prognostic information in NSCLC, but conflicting data requires further prospective validation and patient validation in clinical trials, also suggesting the importance of epigenetic BRCA1 expression [23–25]. As for BRIP1, Rosenthal et al. found that the variant is associated with the risk of colorectal cancer [26]. It was reported that elevated expression level is associated with increased metastasis and shortened survival in LUAD patients [27, 28]. So far, limited results disclosed that the BRIP1 signature was prognostic for tumor stage, grade, and metastasis. Hence, BRCA1 and BRIP1 could be associated biomarkers for the PDL1-positive expression patient selection receiving chemotherapy and immune checkpoint inhibitor combination therapy needs further exploration and validation.

EREG is a member of the epidermal growth factor (EGF) family of peptide growth factors. Deregulated EREG activity contributes to the progression of a variety of malignancies, including non-small-cell lung cancer [29]. Researchers found that NSCLC with KRAS, BRAF, or EGFR mutations overexpressed EREG, and abrogation of such mutations or associated therapeutic inhibitors could downregulate the EREG expression [30]. Studies demonstrated that cancers, including NSCLC, with a higher tumor mutation burden, have a higher likelihood response to PD1/PDL1 blockades [31, 32]. A phase III trial demonstrated that EREG expression can be a predictor for overall survival in oxaliplatin/fluoropyrimidine plus bevacizumab-treated metastatic colorectal cancer patients without RAS and BRAF mutations [33]. High EREG and AREG expressions are a predictive marker for panitumumab therapy benefit on PFS in RAS wild-type advanced colorectal cancer patients [34]. It was reported that EREG-high tumors in lung adenocarcinoma patients have significantly shorter DFS and OS compared to those with EREG-low tumors. EREG expression was associated with age, gender, and smoking status [29, 35]. Elevated EREG expression was also related to pleural involvement positivity, lymphatic permeation positivity, and vascular invasion positivity. Therefore, EREG might be a predictive response biomarker for PDL1-positive expression patients receiving PD1/PDL1 inhibitors, which needs further exploration and validation.

We also conducted transcription factor analysis on these genes and tried to find potential adjuvant therapeutic targets for immune checkpoint inhibitor treatment. Transcription factors can directly bind to specific DNA tracts in gene promoters, which is a key step toward transcriptional activation or repression [36]. Studies revealed that transcription factors can be potential therapeutic targets based on its significance in a variety of biological processes and aberrant activity in human diseases [37]. A number of researches demonstrated that molecules aimed at targeting transcription factors is a promising strategy in cancer treatment [38, 39]. We speculate that some transcription factors we predicted contribute to these differentially expressed genes we identified and might be potential adjuvant therapeutic targets for PD1 and PDL1 immune checkpoint blockade therapy in lung adenocarcinoma. Future work needs to be done to identify the main transcription factor that regulates the gene expression.

Finally, to identify adjuvant biomarkers that is PDL1 related but negative differential expression genes. Although the expression of these genes has no difference compared with the control, but high or low expression has a prognostic value. CCR5 is expressed higher in invasive tumor tissues than noninvasive tissues according to the subclassification of adenocarcinoma [40]. CCR5 can be expressed by several cell types and there is an elevated CCR5 MDSC expression level in patients with NSCLC [41]. Some evidence suggests that CCR5 and its ligands appear to participate in the canonical immune checkpoint response and elevated expression level correlated with a poor prognosis [42]. It was reported that KRAS can regulate FOSL1 expression and it was an independent survival marker for LADC patients with KRAS mutations [43]. And low expression of FOSL1 in the cytoplasm was linked with advanced tumor stage [44]. NAIP is an antiapoptotic protein and the research in NSCLC is limited. NBN is regarded to be involved in DNA double-strand break repair and DNA damage-induced checkpoint activation. There is a report about NBN mutation in lung cancer [45]. NBN polymorphisms may be genetic biomarkers for NSCLC prognosis especially PFS with platinum-based chemotherapy in the Chinese population [46]. Whether this group of biomarkers could help with the patient selection needs further research. And the expression and the function of these biomarkers in NSCLC need further validation and description in the future. At the same time, cellular sublocalization, mutation, and epigenetic modification should be paid attention to.

As we mentioned before, the PDL1 immunohistochemistry evaluation was not available and the mRNA expression level was used to predict the survival time. In this study, we found that median survival time of patients in the PDL1 high-expression group was 701 days. Median survival time in high expression of the identified biomarkers, BRCA1, BRIP1, and EREG, was 690, 701, and 690 days separately. As for median survival time, biomarkers we identified were not superior to PDL1. It might attribute to the limited sample size and needs further large sample validation. Another reason might be because we assess mRNA levels not protein expression levels; future exploration and validation need to focus on protein expression. There are some limitations using immunohistochemistry to detect the candidate biomarkers we identified. First, expression of BRCA1, BRIP1, and EREG was regulated by various mechanisms, such as signaling pathway, transcription factors as we predicted, and epigenetic factors. Individual difference and intratumor heterogeneity should be taken into account. Second, poor uniformity in immunohistochemistry antibodies and different score algorithms may cause the inaccuracy. So, in the future, large-scale investigation and validation need to be done clinically, and new detection methods need to be developed.

## 5. Conclusions

In conclusion, from the functional enrichment analysis of TCGA database, we extracted a list of PDL1-related genes. These genes have the potential to become additional biomarkers for the selection of patients for PD1/PDL1 inhibitors. The predicted transcription factors of these genes might provide adjuvant therapeutic targets and improve the immune therapy responsiveness. In addition, it would be extremely meaningful if the combination of PDL1 and these potential biomarkers have a more accurate indication of patient selection. Further investigation of these genes could lead to novel insights into the immunotherapy for lung adenocarcinoma patients.

## Figures and Tables

**Table 1:** PDL1 coexpressed genes not only differentially expressed in lung adenocarcinoma but also contribute to overall survival.

mRNA	Cor	log_2_foldchange	*P* value
ANLN	0.36109	3.77289	5.92E-05
ARNTL2	0.3383	2.4906	0.00144954
AVL9	0.31527	1.08517	0.04576124
BRCA1	0.3755	1.46278	0.005052667
BRIP1	0.32595	2.68693	0.04478685
CD53	0.37628	-1.0313	0.005429641
CENPE	0.32484	2.65322	0.006552998
CRTAM	0.37113	-1.0706	0.02319598
CSF2RB	0.32911	-1.0044	0.008033928
CYBB	0.46179	-1.3471	0.01465277
DOCK11	0.42634	-1.2654	0.01724113
ELOVL6	0.41838	1.28021	0.01821207
EREG	0.51626	2.58762	0.003137182
FCGR1B	0.346	-1.0663	0.01076134
GIMAP4	0.37656	-1.4853	0.001781643
GIMAP5	0.34985	-1.6241	0.006549764
GPR65	0.37454	-1.1182	0.03443263
GPX3	0.34227	-2.2637	0.009965236
GSG2	0.31185	1.92806	0.04657218
HACD4	0.37455	-1.4402	0.002306662
KIAA1524	0.33152	1.96877	0.01320607
KIF20B	0.34274	1.29505	0.02865572
KRT80	0.32673	2.43778	0.01169369
LPXN	0.33821	-1.1081	0.004965741
LRRC8C	0.33574	-1.12	0.0407523
MELK	0.35309	3.61062	0.01053599
MNDA	0.35278	-1.4709	0.02036211
NCAPG2	0.31191	1.77961	0.003450744
NCKAP1L	0.37919	-1.2778	0.003063421
NGEF	0.31071	3.00327	0.01781168
PLK4	0.32713	2.03832	0.01624677
PMAIP1	0.40395	1.63802	0.03528504
RALGPS2	0.31509	1.65783	0.02929378
RGS20	0.35033	2.9063	0.009297155
SCIMP	0.31898	-1.5645	0.000474133
SHCBP1	0.42305	2.27346	0.001114681
SMCO2	0.33114	2.49326	0.002347819
TLR4	0.35033	-1.5721	0.0217862
TRIM6	0.31406	1.16306	0.01236621

Cor: correlation coefficient; log_2_foldchange: negative number represents downregulation, positive number represents upregulation; *P* value: overall survival of patients in log-rank test.

**Table 2:** Top 10 transcription factor predictions of candidate genes.

BRCA1		BRIP1		EREG	
TF	Motif	TF	Motif	TF	Motif
RFX4	GGTTTCCGTGGCAACG	NFYB	AGCTCGACCAATCAC	E2F6	GGGCGGGAGCA
RFX5	CGTTGCCACGGAAACC	TFDP1	GGGCGGGAGGC	RXRG	GAGTGCACGGGCAGGGCG
RFX5	GGTTTCCGTGGCAACG	CTCFL	GTCGAGGGGGCGGG	RARA	AGTGCACGGGCAGGGCG
RFX4	CGTTGCCACGGAAACC	HAND1	GGACTGGGGC	E2F6	GGGAGGGAGGA
RFX2	CGTTGCCACGGAAACC	SP2	TTCCCGCCTCCCGCC	NR2F1	GTGAGGTCAAGAG
RFX2	GGTTTCCGTGGCAACG	CTCFL	GGACTGGGGCCGCC	NFYB	TCCCCGGCCAATCGG
SOX10	CGGACAAAGAC	SP2	CTCCCGCCCCCTCGA	MEIS2	CTGACAGC
SOX3	TCTTTGTCCG	TEAD2	CCCCAGTCCTGCA	RUNX1	CGCTGGGGCTT
SOX6	TCTTTGTCCG	TFDP1	AGGCGGGAATT	E2F4	GGGCGGGAGCA
TFDP1	GCGCGGGAATT	E2F6	GGGCGGGAGGC	MAFG	GATGAC

TF, transcription factor; motif, DNA sequence of target species which is predicted to be bound by the binding protein.

## Data Availability

The data used to support the findings of this study are included within the article.
